# Baricitinib therapy response in rheumatoid arthritis patients associates to STAT1 phosphorylation in monocytes

**DOI:** 10.3389/fimmu.2022.932240

**Published:** 2022-07-25

**Authors:** Gloria Tucci, Cristina Garufi, Ilenia Pacella, Marta Zagaglioni, Alessandra Pinzon Grimaldos, Fulvia Ceccarelli, Fabrizio Conti, Francesca Romana Spinelli, Silvia Piconese

**Affiliations:** ^1^ Department of Internal Clinical Sciences, Anesthesiology and Cardiovascular Sciences, Sapienza University of Rome, Rome, Italy; ^2^ Unità di Neuroimmunologia, IRCCS Fondazione Santa Lucia, Rome, Italy; ^3^ Laboratory Affiliated to Istituto Pasteur Italia—Fondazione Cenci Bolognetti, Rome, Italy

**Keywords:** interferon, STAT, JAKi, monocytes, rheumatoid arthritis, ISGs

## Abstract

Baricitinib is a Janus kinase (JAK) 1 and 2 inhibitor approved for treating rheumatoid arthritis (RA). The JAK/STAT system is essential in the intracellular signaling of different cytokines and in the activation process of the monocyte lineage. This study verifies the effects of baricitinib on STAT phosphorylation in monocytes of RA patients and evaluates the correlation between STAT phosphorylation and response to therapy. We evaluated the disease activity of patients (DAS28CRP) at baseline (T0) and after 4 and 12 weeks (T1–T3) of treatment with baricitinib, dividing them into responders (n = 7) and non-responders (n = 7) based on the reduction of DAS28CRP between T0 and T1 of at least 1.2 points. Through flow cytometry, STAT1 phosphorylation was analyzed at T0/T1/T3 in monocytes, at basal conditions and after IL2, IFNα, and IL6 stimulation. We showed that monocyte frequency decreased from T0 to T1 only in responders. Regarding the phosphorylation of STAT1, we observed a tendency for higher basal pSTAT1 in monocytes of non-responder patients and, after 4 weeks, a significant reduction of cytokine-induced pSTAT1 in monocytes of responders compared with non-responders. The single IFNα stimulation only partially recapitulated the differences in STAT1 phosphorylation between the two patient subgroups. Finally, responders showed an increased IFN signature at baseline compared with non-responders. These results may suggest that monocyte frequency and STAT1 phosphorylation in circulating monocytes could represent early markers of response to baricitinib therapy.

## Introduction

Rheumatoid arthritis (RA) is a systemic, chronic, autoimmune disease affecting the synovial joints as well as different organs (1). The activation of the innate and adaptive immunity (monocytes, macrophages, mast cells, neutrophils, dendritic cells, T lymphocytes, and B lymphocytes) is responsible for the inflammation of the synovial membrane, which in response determines a switch of synovial cells into fibroblast-like synoviocytes (FLS), responsible for the aggressive inflammatory phenotype of rheumatoid synovitis (1). Monocytes, macrophages, and dendritic cells are mononuclear phagocytes, and their roles in autoimmune and chronic inflammatory diseases, such as RA, are crucial (2, 3). The classification of monocyte subpopulations is based on the expression of the surface markers CD14 and CD16: the classical monocyte population (CD14+ CD16−/low), the intermediate monocyte population (CD14+CD16+), and the nonclassical monocyte population (CD14−/lowCD16+). The proportion of monocytes belonging to the intermediate and nonclassical groups may increase under different pathological conditions and may indicate RA disease activity and response to therapy (4). A previous analysis by Chara et al. evaluated the association between circulating monocyte number and subsets in RA patients treated with adalimumab, showing their predictive value for clinical response after six-month treatment (5).

The Janus-kinase (JAK) family includes JAK1, JAK2, JAK3, and TYK2 receptor-associated tyrosine kinases, able to activate the STAT (STAT1, 2, 3, 4, 5A, 5B, and 6) transcription factors. The JAK-STAT pathway is utilized by several type I and II cytokines, such as interferon (IFN), GM-CSF, and IL-6, that play a pathogenic role in RA (6). JAK-inhibitors are synthetic targeted molecules designed to inhibit the JAK activation pathway. Among the available JAK inhibitors, baricitinib preferentially acts on JAK1/JAK2.

The JAK-STAT pathway is one of the most important pathways for FLS proliferation and cartilage erosion through the secretion of matrix metalloproteinases (MMPs) (7). Few data are available on the effect of JAK inhibitors on FLS: IFNγ-induced adhesion and invasion ability are reduced (8), cell death is accelerated and thickening of the synovium is abrogated (9). Moreover, JAK-STAT is one of the intracellular signaling cascades used in the activation and differentiation process of monocytes; its inhibition with JAKi (tofacitinib and baricitinib) was associated with a surface phenotype similar to non-activated macrophages (Mφ) (10). Moreover, recent evidence seems to suggest that tofacitinib (JAK1/3 and, to a lesser extent, JAK2 inhibitor) can reduce not only the expression of inflammatory but also of fibrotic markers, also inhibiting the differentiation of macrophages toward a profibrotic phenotype (11). Additionally, the migration and mobility capacities of macrophages are also co-linked to JAK-STAT activity, as demonstrated by the altered chemotactic capacity of monocytes after peficitinib treatment (12).

This study verified the effects of baricitinib treatment on STAT phosphorylation in peripheral blood mononuclear cells (PBMCs) of RA patients and evaluated any correlation between STAT phosphorylation status and treatment response.

## Materials and methods

### Patients: inclusion criteria and study design

We enrolled consecutive patients referred to the Rheumatology outpatient clinic of the Sapienza University of Rome (Sapienza Arthritis Center) with a diagnosis of Rheumatoid Arthritis (RA) according to the American College of Rheumatology/European League Against Rheumatism (ACR/EULAR) 2010 criteria (13) from January to December 2018. All patients started baricitinib at 4 mg daily for RA that was moderately to severely active and with an inadequate response or intolerance to ≥1 conventional synthetic Disease Modifying Antirheumatic Drugs (csDMARDs), as for local indications. All patients signed a dedicated informed consent for participation in this study. Before starting baricitinib, all patients were screened for latent tuberculosis, previous hepatitis B/C virus infections, and varicella-zoster. Patients with ongoing or recent infections, a history of malignancy in the past 5 years, or any other condition that contraindicated the initiation of baricitinib were excluded from the study. Baseline demographic data (ethnicity, sex, age) and clinical data were collected: weight, height, body mass index (BMI), duration of disease, positivity for rheumatoid factor (RF) and anti-citrullinated peptide antibodies (ACPA), number of previous csDMARDs, and biological Disease Modifying Antirheumatic Drugs (bDMARDs). The disease activity was assessed at the beginning and then after 4–12–24 weeks of therapy (T0–T1–T3–T6), by the Disease Activity Score 28 (DAS28) calculated using C-Reactive Protein. Additionally, the assessment of disease activity and pain (PhGA) and the patient global assessment (PGA) by the physician were measured using a visual analog scale (VAS 0–100 mm). Patients were considered responders if, after 4 weeks, they had achieved at least a moderate EULAR response (14). For this study, we selected seven consecutive responders and seven consecutive non-responder patients.

### Preparation of human peripheral blood mononuclear cells

Patient blood samples were collected on the same day of treatment. The whole blood was first diluted in PBS 1× at a 1:1 ratio, then gently layered on a volume (equivalent to the starting blood volume) of Lympholyte (Cedarlane) and centrifuged at 870*g* at room temperature for 25 min with low acceleration/brake. PBMCs were collected at the interface between the layers of Lympholyte and diluted blood and washed with PBS 1×. After cell counting, PBMCs were frozen in cryovials in FBS 10% DMSO and kept at −80°C until the experiment day. PBMCs of all patients and at all time points were longitudinally collected and frozen, to be thawed together on the day of the experiment.

### Flow cytometry for pSTATs

On the day of the analysis, PBMCs were thawed at 37°C, counted and seeded in a 96-well plate. Cells were labeled with viability dye for 30 min at room temperature, then with CD127 Alexa Fluor 647 (since this epitope was sensitive to subsequent fixation), followed by cytokine stimulation that was performed for 15 min at 37°C with a cocktail of IL-6 100 ng/ml (PeproTech), IL-2 100 ng/ml (Roche), and IFN-α2b 40.000 IU/ml (Miltenyi Biotec). Then, cells were fixed and permeabilized using Cytofix Fixation Buffer (BD Biosciences) pre-warmed to 37°C and Perm Buffer III (BD Biosciences) pre-cooled to −20°C, according to the instructions of the manufacturer. Finally, intracellular staining was performed with a cocktail of fluorochrome-conjugated antibodies in PBS 0.5% BSA for 30 min at room temperature. The staining was performed according to the following panel: Viability Dye eFluor780 (65-0865-14, Invitrogen), CD127 Alexa Fluor 647 (558598, BD Biosciences), CD4 PerCP-Cy 5.5 (560650, BD Biosciences), CD8 Brilliant Violet 510 (563919, BD Biosciences), CD14 Brilliant Violet 605 (564054, BD Biosciences), FOXP3 Alexa Fluor 700 (56-4776-41, Invitrogen), CD45RA Brilliant Violet 785 (304140, Biolegend), phospho-STAT1 Alexa Fluor 488 (612596, BD Biosciences), phospho-STAT3 Pacific Blue (560312, BD Biosciences), phospho-STAT4 PE (558249, BD Biosciences), and phospho-STAT5 PE-Cy7 (560117, BD Biosciences). In all flow cytometry experiments, cells were acquired on LSR Fortessa (BD Biosciences).

### Flow cytometry for pSTAT1 in monocytes

Thawed cells were labeled with viability dye in PBS for 20 min at 37°C, followed by surface staining for 15 min at room temperature in PBS 2% FBS. After stimulation with IFN-α2b 40.000 IU/ml (Miltenyi Biotec) for 15 min at 37°C, cells were fixed and permeabilized using Cytofix Fixation Buffer (BD Biosciences) pre-warmed to 37°C and Perm Buffer III (BD Biosciences) pre-cooled to −20°C, according to the instructions of the manufacturer. Fc Block (Human TruStain FcX, Biolegend) in PBS was used to prevent the unwanted binding of Fc receptor CD16 antibody. Finally, intracellular staining was performed for 30 min at room temperature in PBS with 0.5% BSA. The following panel was used: Viability Dye Aqua Brilliant Violet 510 (L34957 A+B, Invitrogen), CD3 APC-Cy7 (317341, Biolegend), CD19 APC-eFluor780 (47-0199-42, Invitrogen), CD56 APC-eFluor780 (47-0567-42, eBiosciences), CD14 BB700 (566466, BD Biosciences), CD16 Alexa Fluor 647 (557710, BD Biosciences), HLA-DR PE-CF594 (562304, BD Biosciences), and phospho-STAT1 Brilliant Violet 421 (566238, BD Biosciences).

### Intracellular staining of interferon stimulated genes

For the analysis of the protein levels of selected ISGs (ISG15, PKR, and MX1), PBMCs were thawed at 37°C, counted, and seeded in a 96-well plate, and then analyzed directly or stimulated overnight at 37°C with or without IFN-α2b 40.000 IU/ml (Miltenyi Biotec). After stimulation, cells were labeled with a viability dye for 30 min at room temperature in PBS, followed by surface staining in PBS 2% FBS for 20 min at 4°C. Cells were then fixed and permeabilized using the FOXP3/Transcription Factor Fixation/Permeabilization Buffers (eBioscience) according to the instructions of the manufacturer. Fc Block (Human TruStain FcX, Biolegend) in PBS was used to prevent the unwanted binding of Fc receptor CD16 antibody. Finally, intracellular staining was performed for 30 min at room temperature in Permeabilization Buffer (eBioscience). The flow cytometry panel was the following: Viability Dye eFluor780 (65-0865-14, Invitrogen), CD14 Brilliant Violet 421 (563743, BD Biosciences), MXA Alexa Fluor 488 (AMab237298, Abcam), PKR Alexa Fluor 647 (AMab224921, Abcam), and ISG15 PE (IC8044P, R&D Systems).

### Statistical analysis

Analysis was performed using Flowjo 10.8 and Prism 8.0 (GraphPad Software) for statistical tests and graphical presentations. Data are presented as means ± SD. Two-way ANOVA with Geisser–Greenhouse correction and Sidak’s multiple comparisons test was used to assess intergroup differences in longitudinal samples. A Mann–Whitney test was used to assess differences between subgroups. All the P-values lower than 0.1 are shown. A P-value of <0.05 was considered statistically significant.

## Results

### Dynamics and phosphorylation of STATs in selected immune cell subtypes in responder and non responder patients

Investigating a possible immunological marker of clinical response to baricitinib in RA patients, we longitudinally analyzed the frequencies and the STAT phosphorylation status of four selected immune cell types, i.e., monocytes, CD4 conventional T cells (Tconv), regulatory T cells (Treg), and CD8 T cells, identified according to the gating strategy shown in **Supplementary Figure 1A**. The main characteristics of responder (R = 7) and non-responder (NR = 7) patients are summarized in **Table 1**.

**Table 1:** Clinical and demographic features of enrolled RA patients.

Clinical and demographic features of enrolled patients	Responders n = 7	Non-responders n = 7	*p*
Age (years), mean ± SD	52.77 ± 13.5	59.95 ± 13.18	ns
Female : Male	7:0	5:2	ns
BMI*, mean ± SD	24.03 ± 1.53	24.53 ± 2.38	ns
Disease duration (months), mean ± SD	137 ± 73.6	228 ± 136.12	ns
Rheumatoid Factor, n (%)	4 (57.14)	3 (42.86)	ns
ACPA, n (%)	4 (57.14)	3 (42.86)	ns
DAS28CRP at baseline visit, mean ± SD	5.53 ± 1.4	5.82 ± 1.17	ns
Number of previous bDMARDs, n (%)	3.14 ± 1.86	3.43 ± 1.9	ns
Baricitinib in monoterapy, n (%)	5 (71.42)	3 (42.86)	ns
Daily PDN dose, mean ± SD	2.28 ± 3.86	4 ± 3.62	ns

SD, Standard Deviation; BMI, Body Mass Index; ACPA, anti-citrullinated peptide antibodies; DAS28CRP, Disease Activity Score 28 calculated with C-Reactive Protein; bDMARDs, biological Disease Modifying Antirheumatic Drugs; PDN, prednisone.

At baseline, the frequency of monocytes and CD8 T cells tended to be lower in R patients than in NR patients (n = 4/group); the percentage of monocytes remained lower at all the time points, reaching statistical significance at T3; conversely, Tconv and Treg percentages were similar in R and NR and were not affected by treatment with baricitinib (**Supplementary Figure 2A**).

Then, we assessed the phosphorylation status of multiple STATs (1, 3, 4, and 5) in these immune cell subsets, either unstimulated or briefly exposed to a cytokine cocktail pulse (IL-2, IL-6, and IFNα), to estimate the pre-existing STAT activation, and the responsiveness to a *de novo* stimulation, respectively. Overall, the stimulation increased the phosphorylation of STAT 1, 3, and 5 in all cell subpopulations (while pSTAT4 was only detected at low levels in Tconv and CD8 T cells) (**Supplementary Figure 3**), as also evident in a multidimensionality reduction plot (**Supplementary Figure 4**). When we analyzed the single STAT profiles, we did not find significant differences between R and NR patients at any time point, STAT, or cell type; however, we observed a trend, especially regarding monocytes (**Supplementary Figure 2B**). Indeed, basal STAT1 phosphorylation tended to be lower in monocytes of R patients at all time points; conversely, cytokine-induced STAT1 phosphorylation was slightly reduced from T0 to T1 (but not at T3) in R but not in NR patients (**Supplementary Figure 2B**, upper left panel).

**Figure 1:**
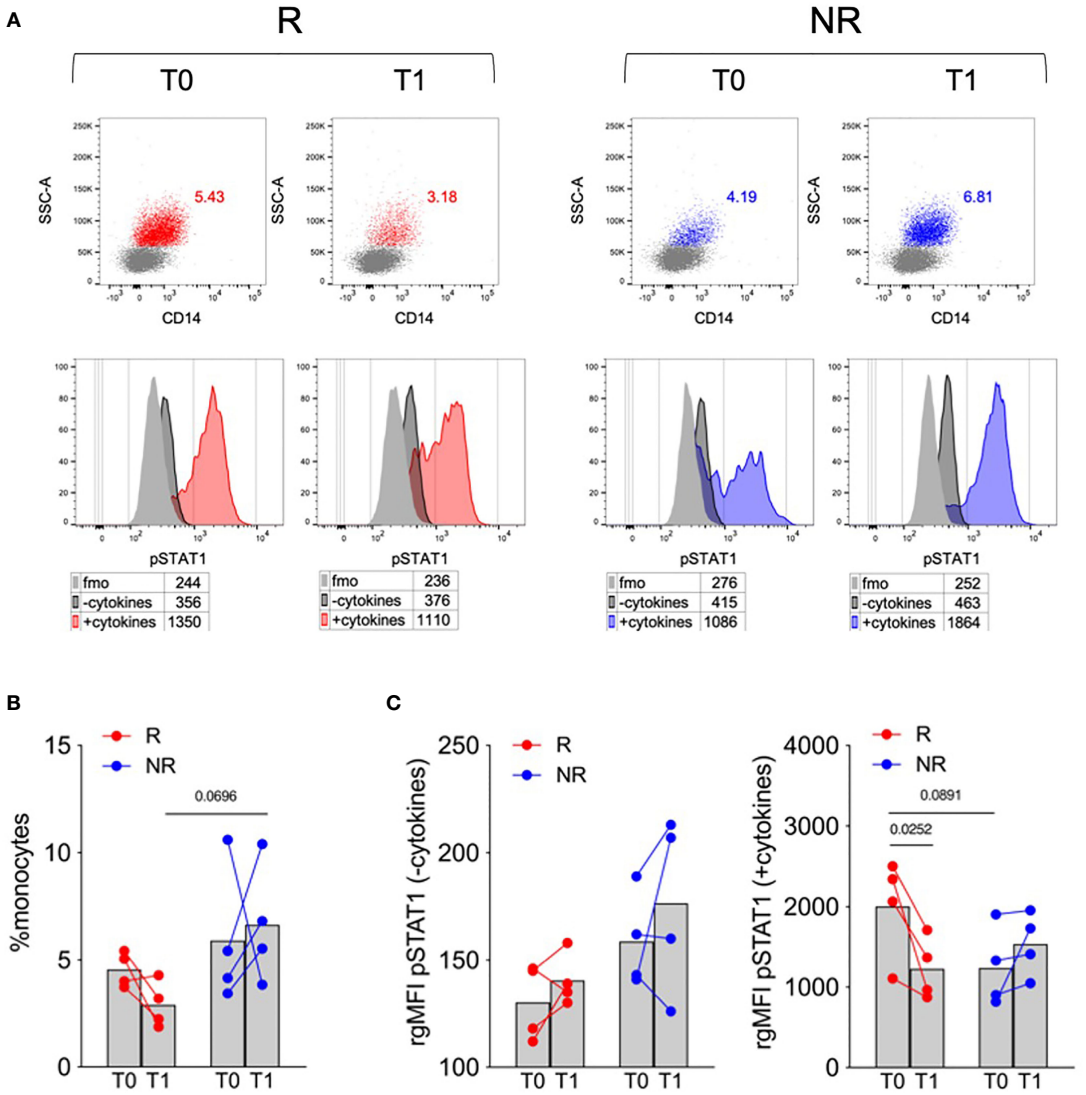
Monocyte frequency and STAT1 phosphorylation in response to a cytokine cocktail in R versus NR patients. **(A)** Representative overlays showing monocytes percentages in PBMCs (upper panels) and STAT1 phosphorylation (pSTAT1) in untreated (−cytokines) or cytokine cocktail-stimulated (+cytokines) monocytes (lower panels) in PBMCs of R and NR patients (n = 4/group), collected before (T0) and after 1 (T1) month post baricitinib therapy starting. Numbers in tables indicate the gMFI of pSTAT1 in each condition; fmo, fluorescence-minus-one control. **(B, C)** Cumulative analysis of monocyte percentage **(B)** and the rgMFI of pSTAT1 in monocytes **(C)**, calculated by subtracting the gMFI of the respective fmo control, in PBMCs of R and NR patients (n = 4/group), collected before (T0) and after 1 (T1) month post baricitinib therapy starting. Bars represent means *P-*values were calculated by 2-way ANOVA with Geisser–Greenhouse correction and Sidak’s multiple comparisons test.

Based on this preliminary screening, we thus focused our analysis on STAT1 phosphorylation at early time points in monocytes, which were identified using a more conservative gating strategy (**Supplementary Figure 1B**). This analysis highlighted that, at T0, samples of R patients displayed a trend towards lower monocyte percentage and higher cytokine-induced STAT1 phosphorylation. Both monocyte percentage and cytokine-induced STAT1 phosphorylation decreased from T0 to T1 only in R and not in NR patients (**Figure 1**). These results suggest that monocyte dynamics and cytokine responsiveness in terms of STAT1 phosphorylation might represent an immune target associated with the clinical response to baricitinib.

### Monocyte frequency and STAT1 phosphorylation after IFNα stimulation decreased in responder patients

**Figure 2:**
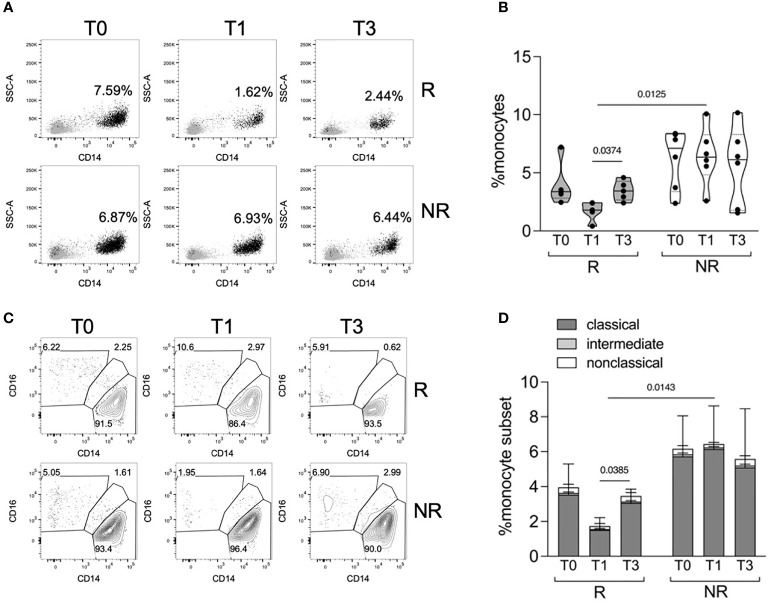
Monocyte frequency and subpopulations in R versus NR patients. **(A, B)** Representative overlays **(A)** and distributions **(B)** of monocyte percentages in PBMCs of R (n = 5) and NR (n = 6) patients, collected before (T0) and after 1 (T1) and 3 (T3) months post baricitinib therapy starting. *P-*values were calculated by 2-way ANOVA with Geisser–Greenhouse correction and Sidak’s multiple comparisons test. **(C, D)** Representative contour plots **(C)** and stacked histograms **(D)** showing the percentage of classical, intermediate, and nonclassical monocytes in PBMCs of R (n = 5) and NR (n = 6) patients at the indicated time points. *P-*values were calculated by 2-way ANOVA with Geisser–Greenhouse correction and Sidak’s multiple comparisons test, comparing classical monocyte frequencies in the indicated conditions.

Recent data showed the influence of increased levels of serum IFNα in predicting the response to TNFα inhibitors in RA patients (15); considering the effect of the cytokine cocktail stimulation on STAT1 phosphorylation in R versus NR patients, we decided to investigate and isolate the effect of IFNα. We therefore focused the subsequent analyses on the characterization of monocyte response to IFNα, in PBMCs collected at T0, T1, and T3 from R and NR patients (n = 5–6/group) (**Table 1**). For this analysis, monocytes were gated using a stringent strategy as CD3− CD56− CD19− HLADR+ CD14+/− CD16+/− live single cells (**Supplementary Figure 5**). When monocyte frequency was analyzed using this strategy, we confirmed that R patients tended to have a lower monocyte frequency at T0 compared with NR, and that only R patients experienced a decrease in monocyte frequency from T0 to T1, partially restored at T3 (**Figures 2A**, **B**). The three subpopulations of classical (CD14+CD16−), intermediate (CD14+CD16+), and nonclassical (CD14−CD16+) monocytes were identified: at all the tested time points, classical monocytes represented most monocytes, and their frequency declined from T0 to T1 in R patients (**Figures 2C**, **D**).

**Figure 3:**
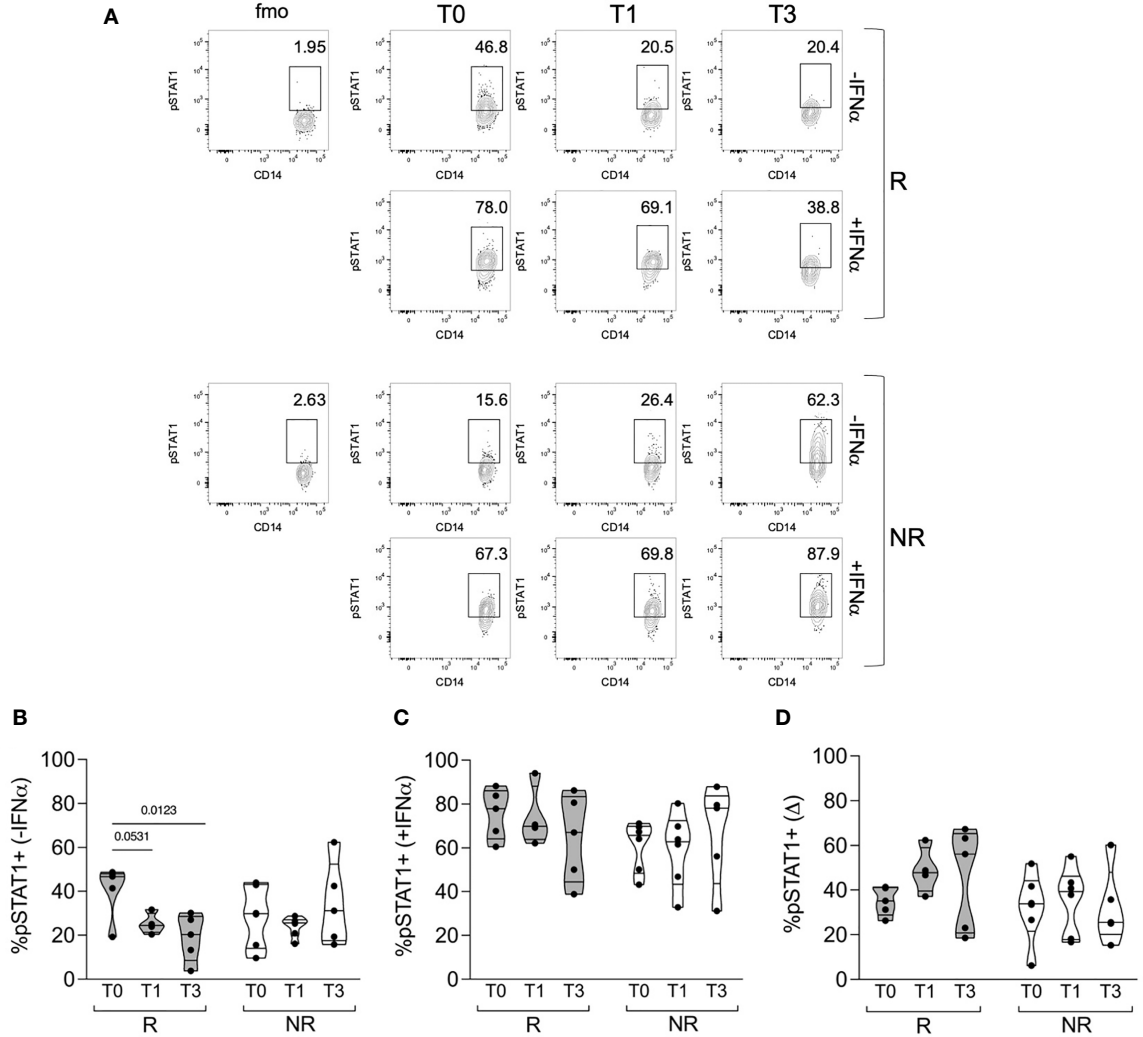
STAT1 phosphorylation in monocytes of R versus NR patients. **(A)** Representative contour plots showing the percentages of pSTAT1+ cells in gated classical monocytes from PBMCs of one R and one NR patient, collected before (T0) and after 1 (T1) and 3 (T3) months post baricitinib therapy starting. Cells were stained after culture for 30 min with (+IFNα) or without (−IFNα) 4 ∗ 10^4^ IU/ml of IFNα. **(B–D)** Cumulative distributions of the percentages of pSTAT1+ cells without IFNα stimulation **(B)** or with IFNα stimulation **(C)**, and of the difference (Δ) between the two values calculated for each sample and condition **(D)**. The analysis was performed in PBMCs of R (n = 5) and NR (n = 6) patients at the indicated time points. Samples were included in the analysis only if enough cells were found in the monocyte gate. *P-*values were calculated by 2-way ANOVA with Geisser–Greenhouse correction and Sidak’s multiple comparisons test.

We then analyzed the percentage of classical monocytes, either unstimulated or shortly pulsed with IFNα, that were positive for phosphorylated STAT1 (**Figure 3A**). Unstimulated cells showed a certain proportion of phosphorylated STAT1 that decreased only in R patients from T0 to T1 and significantly to T3 (**Figure 3B**). After IFNα stimulation, we did not find any significant difference in the percentage of pSTAT1+ cells in R compared with NR patients; however, there was a trend for higher pSTAT1+ monocytes at T0, which was reduced at T3, in the monocytes of R patients compared with NR (**Figure 3C**). Consequently, the difference in pSTAT1+ cell percentage between unstimulated and stimulated cells tended to increase from T0 to T1 and T3 in R but not in NR patients (**Figure 3D**). These data support the idea that the modulation of basal STAT1 phosphorylation in monocytes is associated with the clinical response to baricitinib and that therapy may target the response of monocytes to cytokines besides IFNα.

### Monocytes from responder patients basally express PKR, but not ISG15, at higher levels compared to non-responder patients

**Figure 4:**
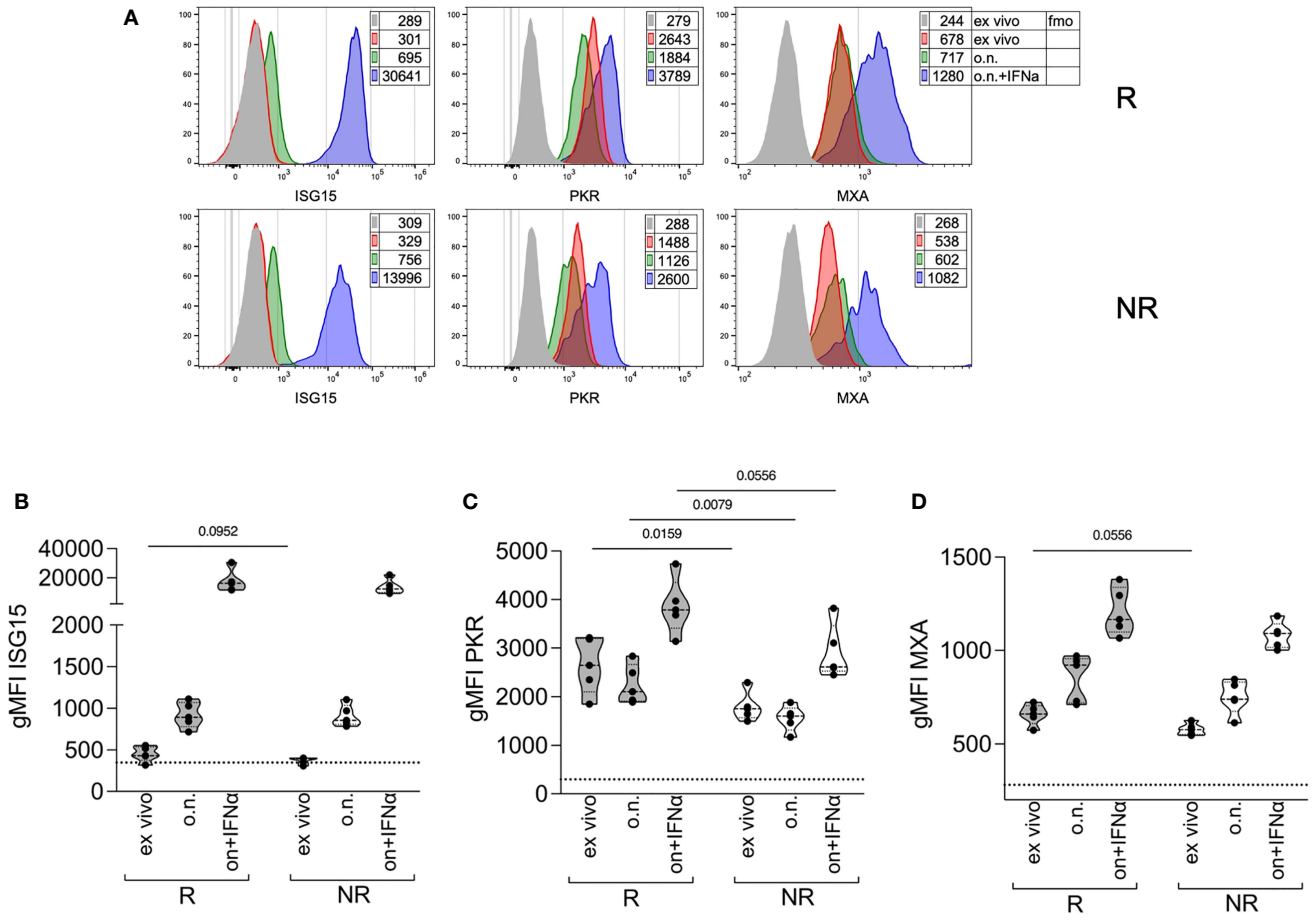
Expression of ISGs in monocytes at therapy starting of R versus NR patients. **(A)** Representative histogram overlays plots showing the expression of ISG15, PKR, and MXA by intracellular staining in gated classical monocytes from the PBMCs of one R and one NR patient collected at baricitinib therapy starting (T0). Gray histograms represent the fmo, fluorescence-minus-one control; red represents cells stained *ex vivo*; green represents cells stained after overnight (o.n.) culture; blue represents cells stained after o.n. culture in the presence of 4 ∗ 10^4^ IU/ml of IFNα. Numbers in tables indicate the gMFI. **(B–D)** Cumulative distributions of the intracellular expression (measured as the gMFI) of ISG15 **(B)**, PKR **(C)**, and MXA **(D**), in gated classical monocytes from PBMCs of R and NR patients (n = 5/group) collected at baricitinib therapy starting (T0), and stained *ex vivo* or after overnight (o.n.) culture in the absence or the presence (o.n. + IFNα) of 4 ∗ 10^4^ IU/ml of IFNα. The dotted lines indicate the average fmo values for each protein. *P-*values were calculated by Mann–Whitney test. .

The data collected so far indicate that R patients had higher STAT1 phosphorylation both basally and in response to IFNα stimulation. IFNα responsiveness can be modulated by the pre-existing IFN signature. We analyzed, through intracellular flow cytometry, the expression of ISG15—an IFNα-negative regulator (16), with two other two ISGs, namely, PKR and MXA, which do not play any known inhibitory role on STAT1. The expression of these genes was analyzed *ex vivo* or after a culture overnight with IFNα, as a positive control. All three proteins were upregulated after *in vitro* culture with IFNα, in monocytes from both R and NR patients (**Figure 4**). ISG15 was slightly higher in monocytes from R patients compared to NR *ex vivo*, but the difference was no more evident after culture, irrespective of IFNα addition (**Figure 4B**). Conversely, PKR expression was significantly higher in monocytes from R patients both *ex vivo* and after culture and tended to be higher in R than in NR after IFNα stimulation (**Figure 4C**). Finally, MXA expression tended to be higher in R compared to NR only at baseline (**Figure 4D**). These results confirm the hypothesis that R patients may have a pre-existing IFN-STAT1 signature in their monocytes, which, however, does not prevent subsequent IFN responses, and which may be a target of baricitinib therapy.

## Discussion

The inhibition of the JAK-STAT pathway in RA treatment has gained increasing interest given the pleiotropic effects of this class of drugs in immune and non-immune cells (16). In this pilot investigation, we focused on the role of JAK-inhibition with baricitinib in circulating monocytes. Our results suggest that monocyte frequency and the IFN signature in circulating monocytes represent early markers of response to baricitinib in RA patients. Indeed, we observed that a higher baseline expression of IFN-related genes and STAT1 phosphorylation characterizes patients who will respond to baricitinib.

The first result of the study highlighted a reduction in the frequency of circulating monocytes after 4 weeks of baricitinib therapy only in responder patients; thus, the early reduction in the percentage of circulating monocytes could be able to discriminate responder patients.

Patients with RA have significantly higher percentages of CD14+CD16+ monocytes than healthy subjects (17). Previous studies have shown that glucocorticoids and TNF-inhibitors can reduce CD14+CD16+ monocytes both *in vivo* and *in vitro* (5, 18). A research aimed at studying the effect of TNF-inhibitors on the percentage of monocytes and their ability to differentiate into osteoclasts showed that these drugs could decrease the frequency of classical monocytes (CD14bright/CD16−) after about 6 months of treatment, possibly contributing to the decrease of erosive damage during therapy with TNF-inhibitors (19). Our data suggested that baricitinib could also have the same effects on classical monocytes: the change in CD14+/CD16− cells mostly accounted for the decrease in the percentage of monocytes detected in responder patients compared with non-responders. In another study, TNF-inhibitors significantly decreased CD14hi/CD16− monocytes compared with placebo, whereas the CD14dim/CD16+ monocyte subclass increased significantly; the authors concluded that the shift toward nonclassical monocytes (CD14dim/CD16+) might reflect the recruitment of this cell type to the inflammatory site, an event associated with reduced disease activity (20). Indeed, CD16bright/CD14+ monocytes showed a correlation with disease activity in RA, as evaluated by clinical indices and ultrasound (21, 22).

We showed a trend in monocyte frequency before starting treatment with baricitinib, with lower levels in R patients compared with NR (not significant). We could not correlate this event with disease activity at baseline, since R and NR patients did not present significantly different disease activity at baseline (data not shown).

Type I IFNs are considered crucial in the activation and differentiation of monocytes, as well as in other innate immune cells, and patients with autoimmune conditions have a peculiar “IFN signature” (23, 24). This study confirmed the effect of baricitinib on STAT1 phosphorylation in circulating monocytes, leading us to hypothesize that STAT1 phosphorylation is influenced by a different monocyte priming in patients who will respond to baricitinib therapy than in those who will not respond. Among the tested cytokines, our data suggest that IFNα contributes to the basal phosphorylation status of STAT1 detected in patients who will respond to baricitinib. Indeed, STAT1 activation is involved in the signaling cascade of all IFNs. Generally, binding of IFNs to their receptors leads to JAK1 and STAT1 engagement and conformational changes to the active form (25, 26).

Both infiltration of IFNγ-producing T lymphocytes (27) and elevated levels of STAT1 and STAT target genes (28) have been observed in rheumatoid synovium, and treatment with DMARDs was able to reduce synovial STAT1 expression only in patients in whom the treatment was effective (29). These data confirm the evidence that the IFN/JAK1-2/STAT1 signaling pathway is involved in rheumatoid synovial inflammation.

The IFN signature, i.e., the set of genes induced by IFN, plays a crucial role in RA pathogenesis, even from the earliest stages of the disease onset. In a study on patients with inflammatory arthralgia, naïve to any treatment, the authors demonstrated that patients who would develop RA had a specific IFN signature, with an up-regulation of SIGLEC1 and MS4A4A, differently from patients who presented with non-inflammatory arthralgia (30). Moreover, the authors showed that the genes MSA4A, PDZK1IP1, and EPHB2, at baseline and follow-up, were able to discriminate patients with RA from healthy controls, assuming a predictive value for RA development (30). In established RA, IFN-related genes influence different cell functions such as apoptosis, gene transcription, protein degradation, Th2 induction, and B lymphocyte proliferation (31). Castañeda-Delgado et al. evaluated the different expression of IFN-related genes in different disease stages, correlating their levels with the production of ACPAs. They enrolled patients with RA in the early stages and in established disease, comparing them with patients at high risk of developing RA according to the positivity of ACPA. Those patients with definite diagnosis and/or long-standing disease showed an increased expression of ISG15 compared with early RA patients. In addition, the IFN signature was significantly correlated with the positivity for ACPA and anti-carbamylated protein antibodies (32).

ISG15 is a member of the ubiquitin family, involved in the regulation of various cellular activities, including protein stability, intracellular trafficking, cell cycle control, and immune modulation. ISG15 and downstream members of ISG15 activation are strongly induced by type I IFN and exert negative feedback on the IFN pathway (33). ISG15 protects Tconv cells from STAT1 phosphorylation and induces resistance to IFNα-mediated depletion in Tregs, exerting negative feedback, which can be explained by a desensitizing effect from IFN stimulation (16). Less is known about ISG15 and monocytes in RA. Our results showed that IFNα could induce ISG15 in the whole cohort without a significant difference between responders and non-responders.

To the best of our knowledge, no previous studies have evaluated the effects of JAK inhibitors on a pre-existing IFN signature and on IFN-induced modification of STAT1 phosphorylation in patients with RA. A phase 2 study with baricitinib in patients with systemic lupus erythematosus (SLE) showed a significantly higher expression of the STAT1 gene in SLE patients who had active disease at study entry. Additionally, significant overexpression of STAT1 coupled with STAT2 at baseline correlated with IFN predominance (34). In these patients, treatment with baricitinib reduced mRNA expression of the target genes of transcription factors STAT2 and STAT4, but not STAT1 (34). This analysis was restricted to the baseline IFN genes and did not mention the impact of baricitinib on STAT phosphorylation and IFN-related genes.

In our cohort, the greatest effect of baricitinib in terms of STAT phosphorylation was in STAT1, especially in monocytes.

Some literature data confirm different utilization of STATs in different immune cell subpopulations, supporting the role of STAT1 in monocytes (35). Previous work showed that circulating monocytes—and to a lesser extent, lymphocytes—from patients with RA exhibit higher levels of STAT1, correlating with disease activity (36).

In a recent work on patients with RA (n = 29) and Systemic Sclerosis (n = 21), the former showed a higher baseline level of STAT1 and STAT3 phosphorylation in CD3+ T cells and monocytes compared with healthy controls (37). The authors also investigated the effects of peficitinib, tofacitinib, and baricitinib on STAT phosphorylation induced by IL-2, IL-4, IL-6, IL-13, and IFN in T CD3+ lymphocytes and monocytes, showing that all JAKi were able to suppress STAT phosphorylation downstream of the different cytokines tested (37). Consistent with the results of the study by Kitanaga et al., our data confirmed that baricitinib exerts an inhibitory effect on STAT1 phosphorylation in monocytes stimulated with IL-2, IL-6, and, above all, IFNα.

In our work, STAT1 phosphorylation in monocytes differentiated responder and non-responder patients; after only 4 weeks, monocytes of responder patients were less responsive—in terms of STAT1 phosphorylation—to cytokine stimulation; in contrast, in non-responders, the extent of STAT1 phosphorylation was similar to that observed before therapy. We could therefore suggest that higher baseline STAT1 phosphorylation and the decrease in pSTAT1+ monocytes could be considered an early predictive marker of response to therapy. A previous study suggested an association between the phosphorylation of STAT6 and STAT1 in circulating leukocytes and the response to RA treatment (38). Kuuliala et al. analyzed the intracellular phosphorylation of STAT1 and STAT6 in response to IFNγ and IL-4 in RA patients treated with csDMARDs or bDMARDs (38). At baseline, in patients with treatment-naive RA, IFNγ-induced lymphocyte pSTAT1 and IL-4-induced monocyte pSTAT6 levels were higher in patients who achieved a good response to therapy than in non-responders to bDMARDS (38).

Monocytes are the peripheral blood counterparts of tissue synovial macrophages, the principal immune cell type infiltrating the inflamed synovia. In the tissue, FLS act as effectors, as they are responsive to the inflammatory local milieu, principally by TNF and IFN, transforming into pro-inflammatory behavior. *In vitro* studies have demonstrated that two JAK inhibitors, peficitinib and tofacitinib, suppress the FLS expression of apoptosis-resistant genes, inhibit the production of matrix metalloproteinases, and promote FLS death (9, 39). The T and B cells, macrophages and mast cells infiltrating the synovial tissue are possibly affected by the inhibition of the JAK-STAT pathway too. There is a crucial crosstalk between FLS and immune cells; *in vitro*, JAK inhibitors (tofacitinib, baricitinib, and upadacitinib) can suppress the secretion of different cytokines produced by FLS-stimulated T helper cells, as well as suppress T-cell proliferation, disrupting the circuits in the bidirectional interplay between FLS and immune cells (40). Tofacitinib and ruxolitinib suppress STAT1 activation in TNF-stimulated RA synovial macrophages (41). Moreover, tofacitinib can inhibit the expression of MMPs and interferon-regulated genes, in accordance with the reduction of pSTAT1 and pSTAT3 phosphorylation, supporting the role of IFNγ and IL-6 inhibition (42).

The results of this pilot study suggest that, with a decrease in monocyte frequency, the baseline cytokine (mostly IFNα)-induced phosphorylation of STAT1 could represent an early predictor of treatment response in RA patients starting baricitinib.

## Data availability statement

The raw data supporting the conclusions of this article will be made available by the authors, without undue reservation.

## Ethics statement

This study was reviewed and approved by the Ethical Committee of Policlinico Umberto I—Sapienza University of Rome. The patients/participants provided their written informed consent to participate in this study.

## Author contributions

GT, CG, FS, and SP conceived the project, designed the protocol, wrote the draft, and analyzed the data. IP, MZ, APG, and FCe contributed to study design and monitored experimental procedure and analyzed the data. FCo, FCe, FS, and SP wrote the statistical analysis plan and analyzed the data, supervised the draft and revised the paper. All authors listed have made a substantial, direct, and intellectual contribution to the work and approved it for publication.

## Conflict of interest

CG, FCo and FS received speaker fees from Eli Lilly.

The remaining authors declare that the research was conducted in the absence of any commercial or financial relationships that could be construed as a potential conflict of interest.

## Publisher’s note

All claims expressed in this article are solely those of the authors and do not necessarily represent those of their affiliated organizations, or those of the publisher, the editors and the reviewers. Any product that may be evaluated in this article, or claim that may be made by its manufacturer, is not guaranteed or endorsed by the publisher.
