# Supplementary material for: Baricitinib therapy response in rheumatoid arthritis patients associates to STAT1 phosphorylation in monocytes
Source: Front Immunol. 2022 Jul 25;13:932240. doi: 10.3389/fimmu.2022.932240 (PMC9357974; doi:10.3389/fimmu.2022.932240)
Supplement: Supplementary file 1 [file DataSheet_1.docx]

Baricitinib therapy response in rheumatoid arthritis patients associates to STAT1 phosphorylation in monocytes

**Supplemental figures**


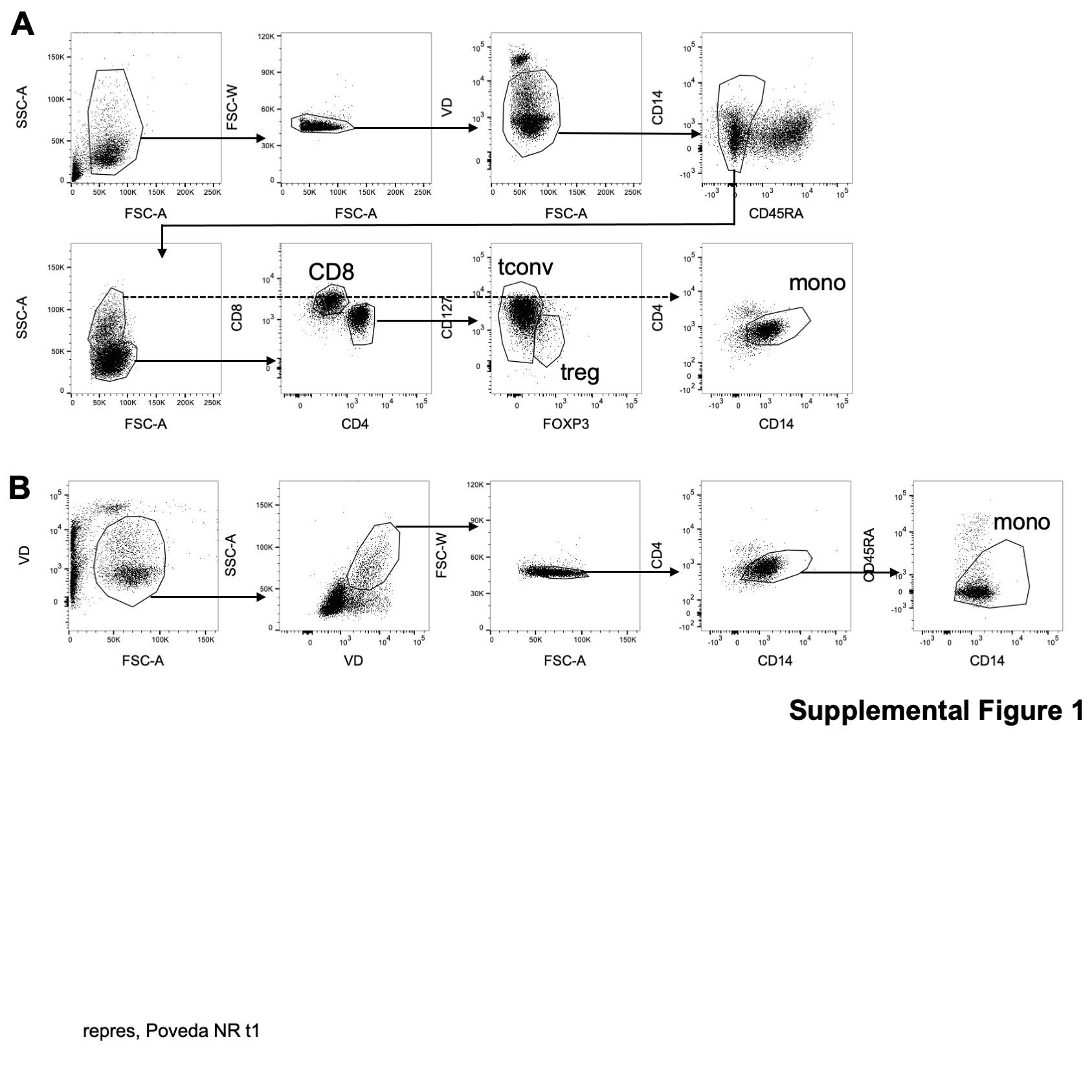


**Supplemental Figure 1. Gating strategy for the analysis of STAT phosphorylation in response to a cytokine cocktail.**

(A) Dot plots showing the sequential gating strategy for the identification of monocytes, Tconv, Treg, and CD8 T cells. Following doublet exclusion, live (viability dye, VD, negative) cells were gated, and CD45RA^+^ cells were excluded. Then, lymphocytes and monocytes were identified based on FSC/SSC parameters. In the monocyte gate, monocytes were identified as CD14+ CD4low cells. In the lymphocyte gate, CD4+ and CD8+ T cells were identified; then, within the CD4+ population, Treg (Foxp3+ CD127low) and Tconv (Foxp3- CD127high) were gated. (B) Dot plots showing the sequential gating strategy specific for the identification of monocytes. VD, viability dye.

**
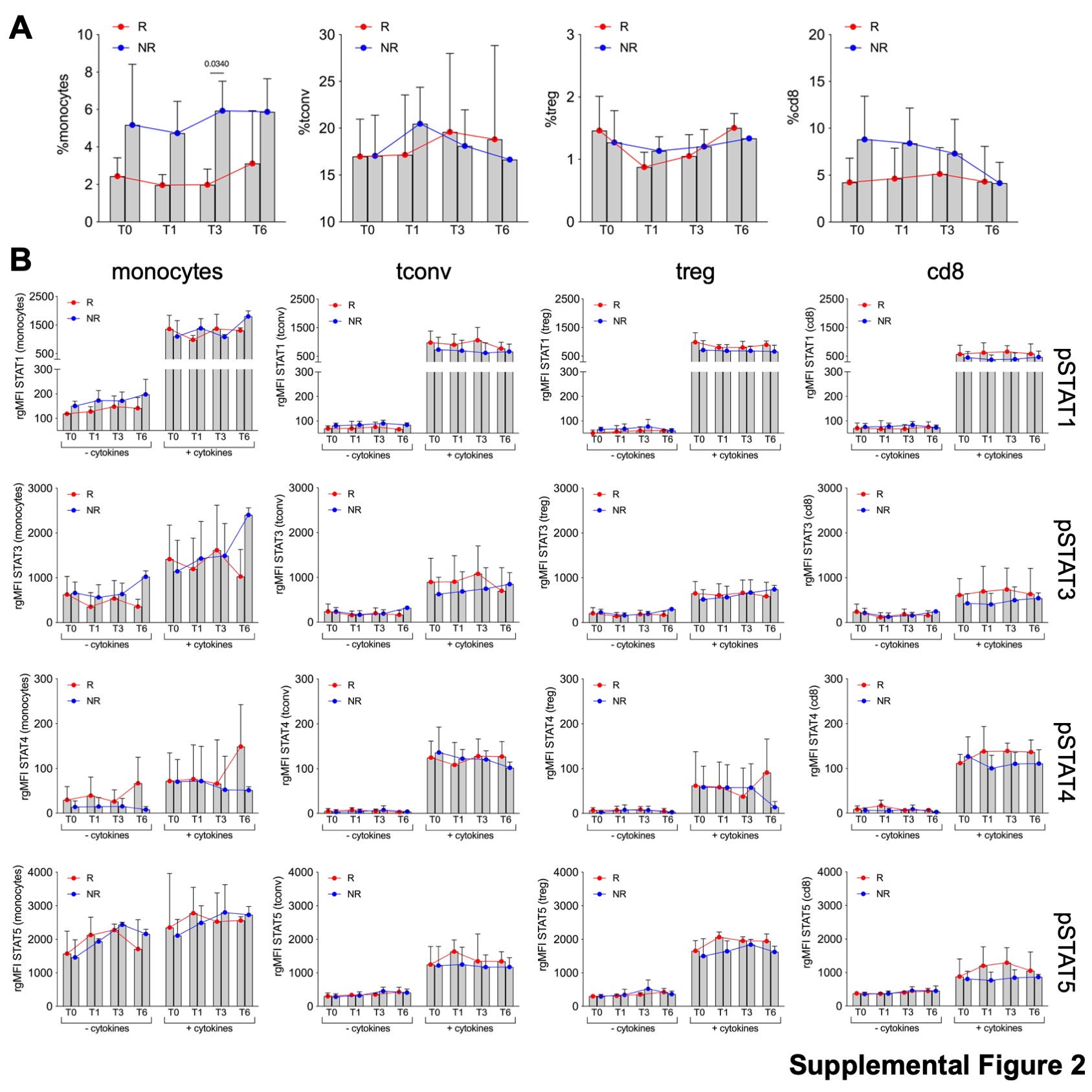
Supplemental Figure 2. Dynamics of cell frequencies and phosphorylation of several STATs in response to a cytokine cocktail in R versus NR patients.**

(A)Frequency of monocytes (CD14+), Tconv (CD4+FOXP3-), Treg (CD4+FOXP3+CD127-), and CD8 T cells (CD8+ CD4-) was analyzed by flow cytometry in PBMCs of R and NR patients (n=4/group), collected before (T0) and after 1 (T1), 3 (T3), and 6 (T6) months post baricitinib therapy starting. Bars represent means and SD. *P* values were calculated by 2-way ANOVA with Geisser-Greenhouse correction and Sidak’s multiple comparisons test.

(B)PBMCs, collected as described above, were left untreated (-cytokines) or were stimulated for 15 minutes with a cytokine cocktail composed of IL-2, IL-6 and IFNα (+cytokines), then combined surface and intracellular staining for phosphorylated STAT1 (pSTAT1), pSTAT3, pSTAT4, and pSTAT5, was performed. The plots show, in gated monocytes, tconv, treg and cd8, the relative geometric mean fluorescence intensity (rgMFI) of each pSTAT, calculated by subtracting the gMFI of the respective fluorescence-minus-one (fmo) control, to normalize the expression between the different cell types. Bars represent means and SD.

**
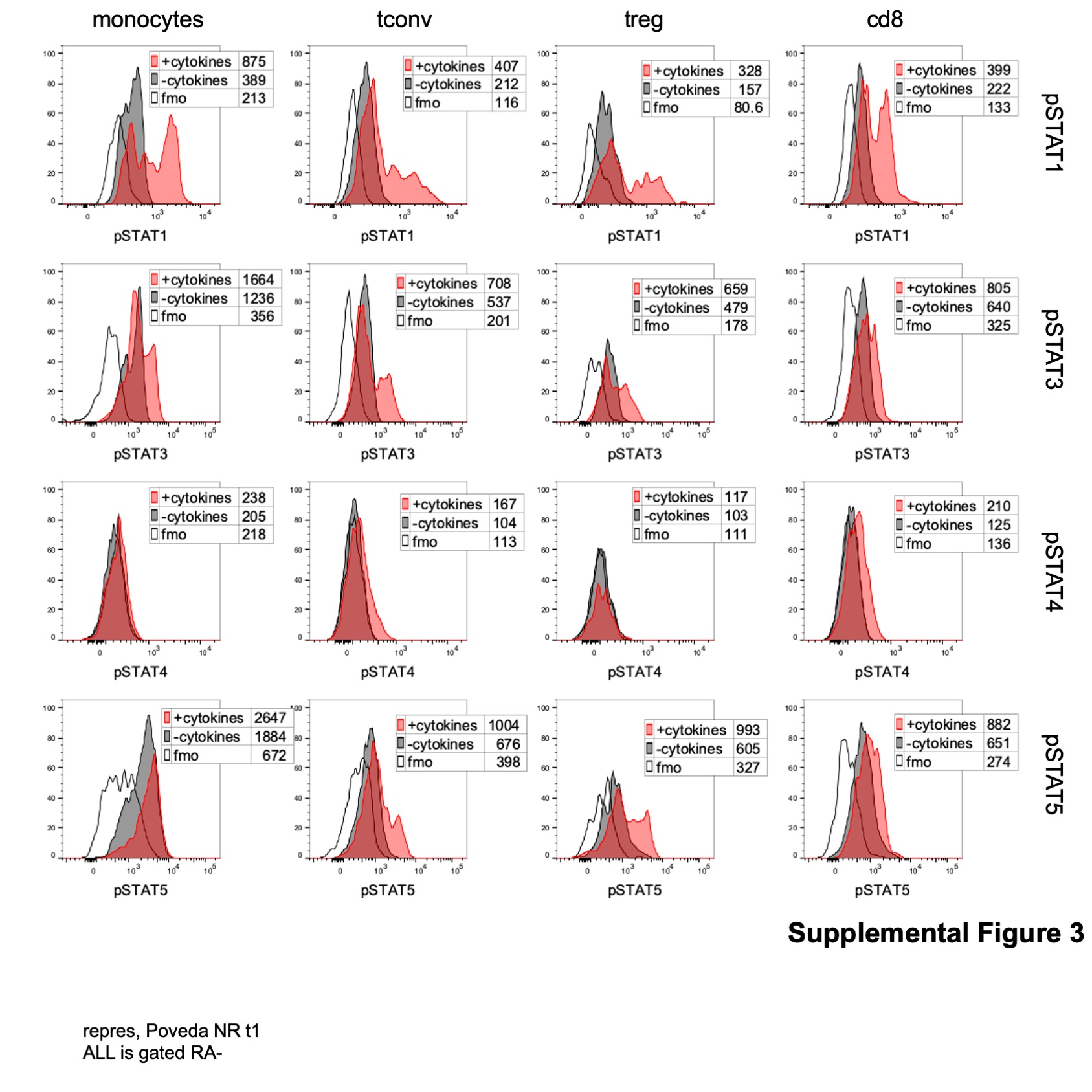
Supplemental Figure 3. Phosphorylation of several STATs in selected cell subsets.**

Representative histogram overlays showing the intracellular content of phosphorylated STAT1, STAT3, STAT4 and STAT5, in gated monocytes, Tconvs, Tregs, and CD8 T cells, either untreated (-cytokines, black) or following a stimulation for 15 minutes with a cytokine cocktail composed of IL-2, IL-6 and IFNα (+cytokines, red). The empty histograms represent the respective fluorescence-minus-one (fmo) controls. Numbers in tables indicate the gMFI.

**
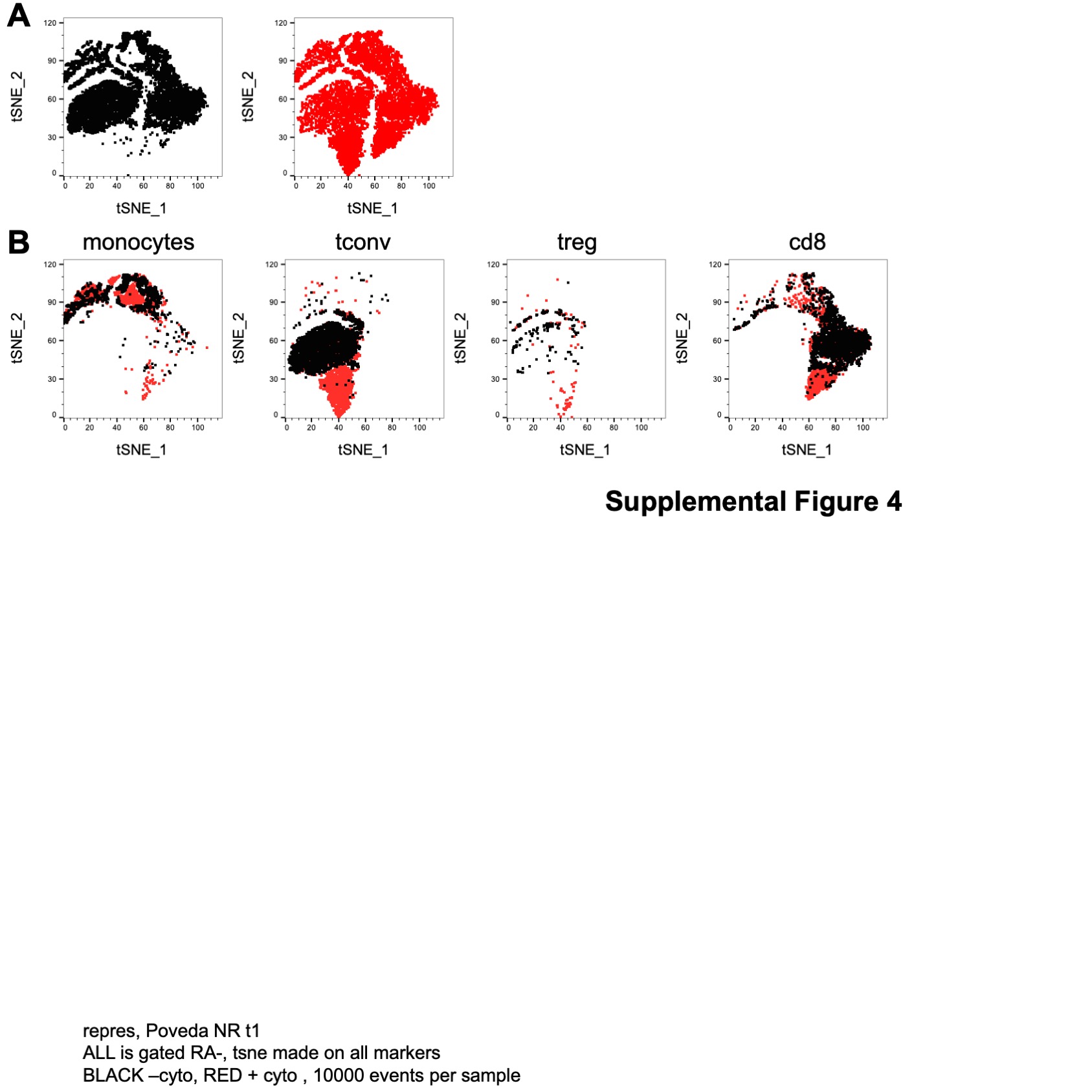
Supplemental Figure 4. Overview of the phosphorylation of several STATs in response to a cytokine cocktail.**

(A) tSNE plots showing the comparison of cell distribution between cells untreated (black) or treated with a cytokine cocktail (red), in PBMCs from a representative patient.

(B) The indicated cells subsets were manually gated and gates were applied to the tSNE distribution. The overlay between untreated (black) and cytokine-treated (red) conditions are shown for each cell subset.


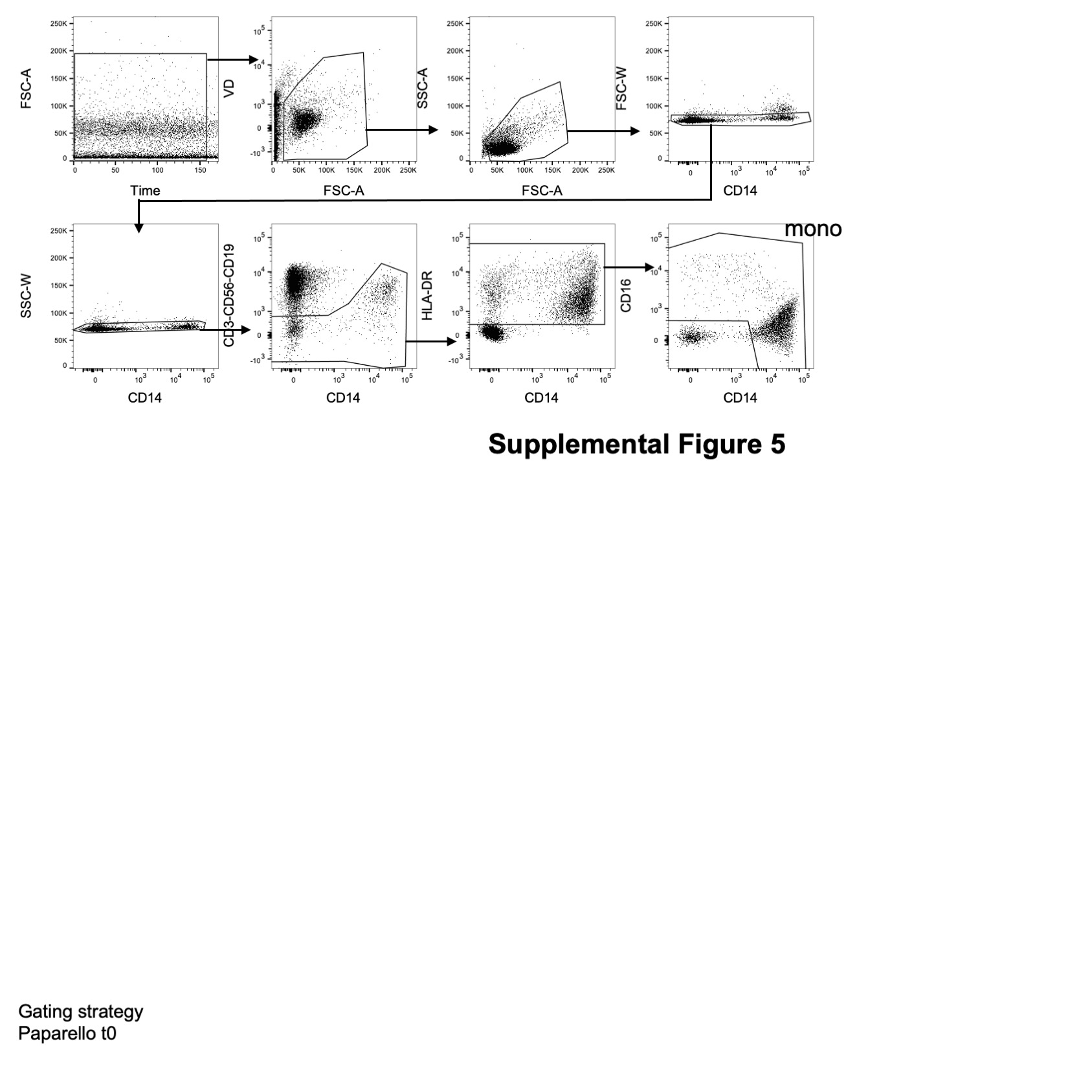


**Supplemental Figure 5. Gating strategy for the analysis of monocyte subsets and STAT1 phosphorylation in monocytes.**

Dot plots showing the sequential gating strategy for the identification of monocytes. VD, viability dye.
